# [Retracted] MicroRNA‑383 suppresses cell proliferation and invasion in colorectal cancer by directly targeting paired box 6

**DOI:** 10.3892/mmr.2023.13055

**Published:** 2023-07-17

**Authors:** Fei Yan, Zhiquan Tu, Li Duan, Dexing Wang, Feng Lin

Mol Med Rep 17: 6893–6901, 2018; DOI: 10.3892/mmr.2018.8682

Following the publication of this paper, it was drawn to the Editor's attention by a concerned reader that certain of the cell migration and invasion assay data shown in Figs. 2C and 5C were strikingly similar to data appearing in different form in other articles by different authors at different research institutes, several of which have been retracted. Owing to the fact that the contentious data in the above article were already under consideration for publication, or had already been published, prior to its submission to *Molecular Medicine Reports*, the Editor has decided that this paper should be retracted from the Journal. The authors were asked for an explanation to account for these concerns, but the Editorial Office did not receive a reply. The Editor apologizes to the readership for any inconvenience caused.

